# The Non-Structural NS1 Protein Unique to Respiratory Syncytial Virus: A Two-State Folding Monomer in Quasi-Equilibrium with a Stable Spherical Oligomer

**DOI:** 10.1371/journal.pone.0074338

**Published:** 2013-09-10

**Authors:** Esteban Pretel, Gabriela Camporeale, Gonzalo de Prat-Gay

**Affiliations:** Protein Structure-Function and Engineering Laboratory, Fundación Instituto Leloir and IIBBA-CONICET, Buenos Aires, Argentina; National Institute for Medical Research, Medical Research Council, London, United Kingdom

## Abstract

Human respiratory syncytial virus (hRSV) is a major infectious agent that cause pediatric respiratory disease worldwide. Considered one of the main virulence factors of hRSV, NS1 is known to suppress type I interferon response and signaling, thus favoring immune evasion. This, together with the fact that NS1 is unique to hRSV among paramyxoviruses, and that has no homology within databases, prompted us to investigate its conformational stability, equilibria and folding. Temperature cooperatively induces conformational changes leading to soluble spherical oligomers (NS1SOs) with amyloid-like or repetitive ß-sheet structures. The onset of the thermal transition is 45°C, and the oligomerization rate is increased by 25-fold from 40 to 46°C. Conformational stability analyzed by chemical perturbation of the NS1 monomer shows a two-state, highly reversible and cooperative unfolding, with a denaturant midpoint of 3.8 M, and a free energy change of 9.6±0.9 kcal⋅mol^−1^. However, two transitions were observed in the chemical perturbation of NS1SOs: the first, from 2.0 to 3.0 M of denaturant, corresponds to a conformational transition and dissociation of the oligomers to the native monomer, indicating a substantial energy barrier. The second transition (2.0 to 3.5 M denaturant) corresponds to full unfolding of the native NS1 monomer. In addition, different cosolvent perturbations converged on the formation of ß-sheet enriched soluble oligomeric species, with secondary structure resembling those obtained after mild temperature treatment. Thus, a unique protein without homologs, structure or mechanistic information may switch between monomers and oligomers in conditions compatible with the cellular environment and be potentially modulated by crowding or compartmentalization. NS1 may act as a reservoir for increased levels and impact on protein turnover.

## Introduction

Human Respiratory Syncytial Virus (hRSV) is the main etiologic agent of severe lower respiratory tract disease, such as bronchiolitis and pneumonia, among infants worldwide. hRSV is also an important cause of morbidity and mortality in immunocompromised subjects and in the elderly [1]. However, there are neither effective vaccines nor specific antivirals available [2]. A monoclonal antibody, Palivizumab, is used only for prevention in high-risk infants and Ribavarin, an antiviral used for treatment of severe illness, has a questionable benefit [3].

hRSV is a negative-strand RNA paramyxovirus, which belongs to the Pneumovirus genus. Its genome of 15,222 nucleotides encodes 10 major subgenomic mRNAS and 11 proteins, including three proteins which form the nucleocapsid. (N, P and L), three transmembrane envelope glycoproteins (F, G and SH), the matrix M protein, the M2-1 protein as the anti-terminator and processivity factor, and the M2-2 protein, which may play a role in switching from RNA transcriptation to RNA replication. The last two remaining proteins, NS1 and NS2, are non-structural proteins that are not present in the mature virion particle [1].

The NS proteins are unique to hRSV, without sequence homologs whatsoever in any other virus or eukaryotic genomes. These are the major viral Interferon (IFN) antagonists and have been implicated in blocking the IFN induction and response pathways [4], [5], [6], [7], [8], [9]. Different anti-interferon molecules are present in other paramyxovirus, such as the V proteins of most Paramyxovirinae, or the C proteins in some respiroviruses (Sendai virus) and morbiliviruses (Measles virus) [10], [11]. The Paramyxovirinae V proteins are translated from edited P gene transcripts whereas C proteins are expressed from P mRNAs by alternative translation initiation. Interestingly, RSV neither edits RNA nor produce proteins corresponding to V or C. Although NS1 and NS2 are not essential for viral replication in vitro, recombinant RSVs with NS1 and NS2 genes deleted are attenuated in vivo and are not able to block IFN induction and signalling, showing a higher IFN expression than wild type viruses [12], [13], [14]. However, deletion of both NS genes results in a higher IFN expression than single deletion of either non-structural protein. Recently, NS2 proteins were shown to bind RIG-I (the helicase sensor) and disrupt downstream signalling [15]. NS1 was reported to co-localize with the mitochondrial signalling protein (MAVS) associated to the mitochondria, inhibiting MAVS-RIG-I interaction and decreasing protein levels of members of the IFN induction pathway as TRAF3 and IKKε [16], [17]. Altogether, this results in a reduction of IFN production.

In connection to the antiviral response pathway, it has been demonstrated that NS proteins target the signal transducer and activator of transcription 2 (STAT2) and mediate its proteosomal degradation; however, the mechanism by which NS proteins produce this effect and the specific role of each protein are not clear. In addition, these proteins play some IFN independent roles. Both NS1 and NS2 prevent early apoptosis in an IFN-independent manner and suppress maturation of dentritic cells and the T lymphocyte response [18], [19], [20].

NS1 is a 15.5 KDa protein, where a recombinant monomeric species was previously described and briefly characterized [21]. In addition, it was hypothesized that NS proteins (NS1 and NS2) can form homo- and heteromers that could govern their intracellular localization ([16], [22]). The same authors identified the cellular microtubule-associated protein 1B (MAP1B) associated to NS1 and NS2 and proposed that this interaction may be important to dock these proteins to other host structures. It has also been shown that NS1 interacts with M and the C-terminus of P viral proteins [23]. Proteomic studies indentified a long list of interacting cellular proteins that are regulated by or interact with NS1, in particular, molecules related to the mitochondria [24], [25], [26].

In this work, we present a comprehensive characterization of one of the principal virulence factors of hRSV, the NS1 protein, directly involved in immune evasion by the virus, a fact that hampers the development of effective vaccines. We set out to study the NS1 protein stability, folding and conformational equilibria, as there is little or no information about its conformational properties and no structure available. Combining different spectroscopic techniques, equilibrium measurements and atomic force microscopy, we describe the conformational plasticity and heterogeneity of this important polypeptide, knowledge that constitutes the biochemical foundations for understanding NS1 interactions, cellular localization and turnover.

## Results

### pH Perturbation of NS1 Monomer Leads to Soluble Oligomers

As the starting point for the analysis of the conformational equilibria and stability of NS1, it was necessary to ascertain unequivocally the species and reference controlled conditions under which the study would be carried out. For purification to homogeneity and storage we used 10 mM Tris buffer (pH 8) containing 0.2 M of NaCl and 1 mM DTT. Unless otherwise stated, measurements were performed in 10 mM Tris buffer (pH 8), 1 mM DTT. Based on this, a size exclusion chromatography (SEC) coupled to static light scattering (SLS) indicated that NS1 was a monomeric protein, determined both from the elution volume and the protein molecular weight. In addition, dynamic light scattering (DLS) showed a monodisperse species with a 4.2±1.2 nm diameter, compatible with a globular monomer, and with a molecular weight of 17.4±1.6 KDa as judged by SEC-SLS (not shown), in agreement with the molecular weight expected from the sequence.

**Figure 1:**
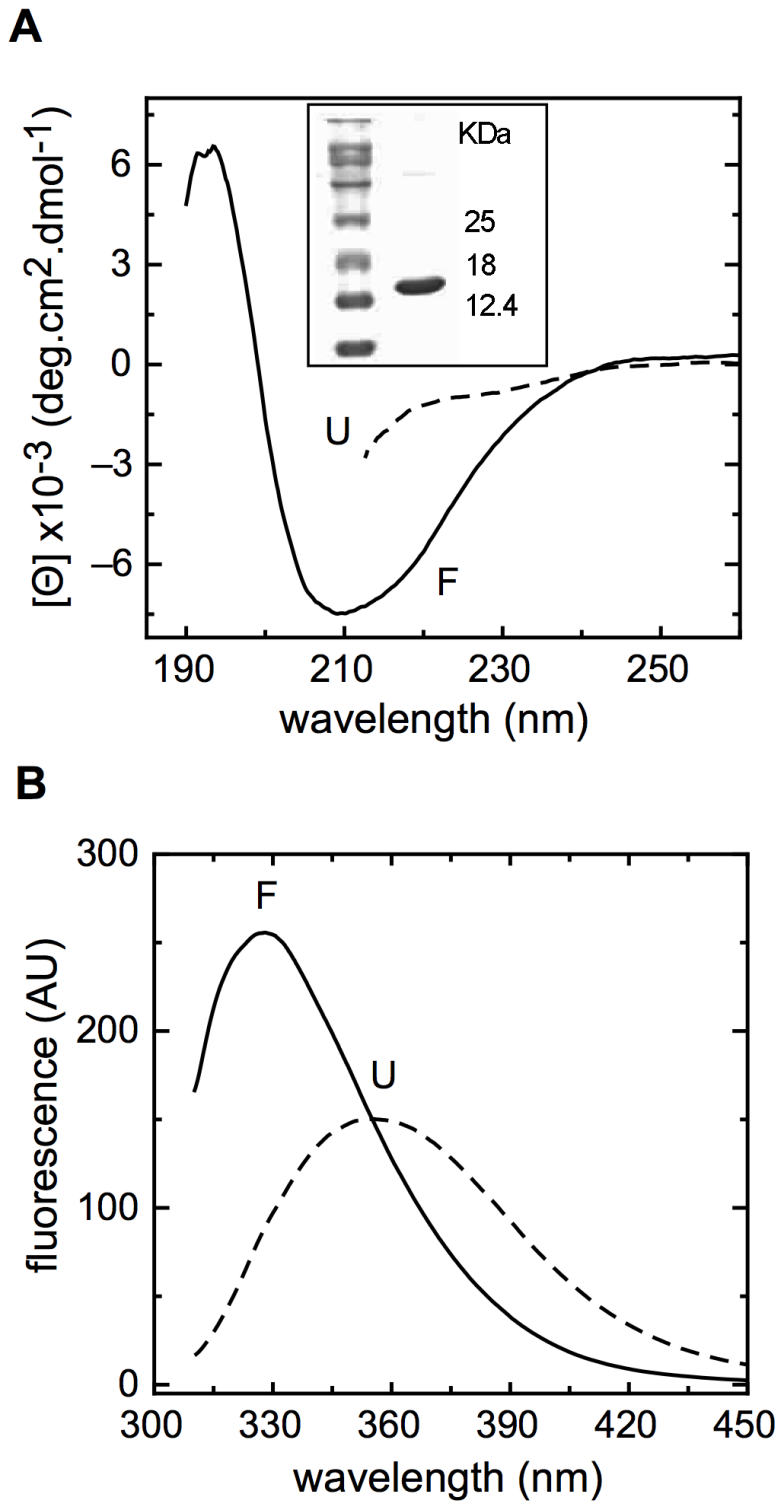
CD and Fluorescence spectra of NS1. (A, B) Far-UV CD spectra and fluorescence spectra of folded and unfolded NS1 (full and dashed lines, respectively). The inset shows purified recombinant protein in a 15% SDS PAGE. The buffer used was Tris 10 mM pH 8 and 1 mM DTT and the same buffer with 6 M Gdm.Cl was used for unfolding experiments. Protein concentration was 10 µM.

Far-UV circular dichroism is consistent with a secondary structure composed of both α-helix and ß-sheet, as previously reported (Fig. 1A, full line, [21]); in addition, the spectrum of the protein in 6 M Gdm.Cl is consistent with an unfolded state (Fig. 1A, dashed line). The fluorescence spectrum showed a maximum at 326 nm (Fig. 1B full line), corresponding to a center of spectral mass (CSM) of 29.297 cm^−1^, while the spectra in 6.0 M Gdm.Cl displayed a maximum shifted to higher wavelengths, corresponding to a center of spectral mass of 27,405 cm^−1^, as expected for a tryptophan exposed to the solvent. This result indicates that the protein single tryptophan residue is buried from the solvent, constituting an excellent probe for the studies proposed.

**Figure 2:**
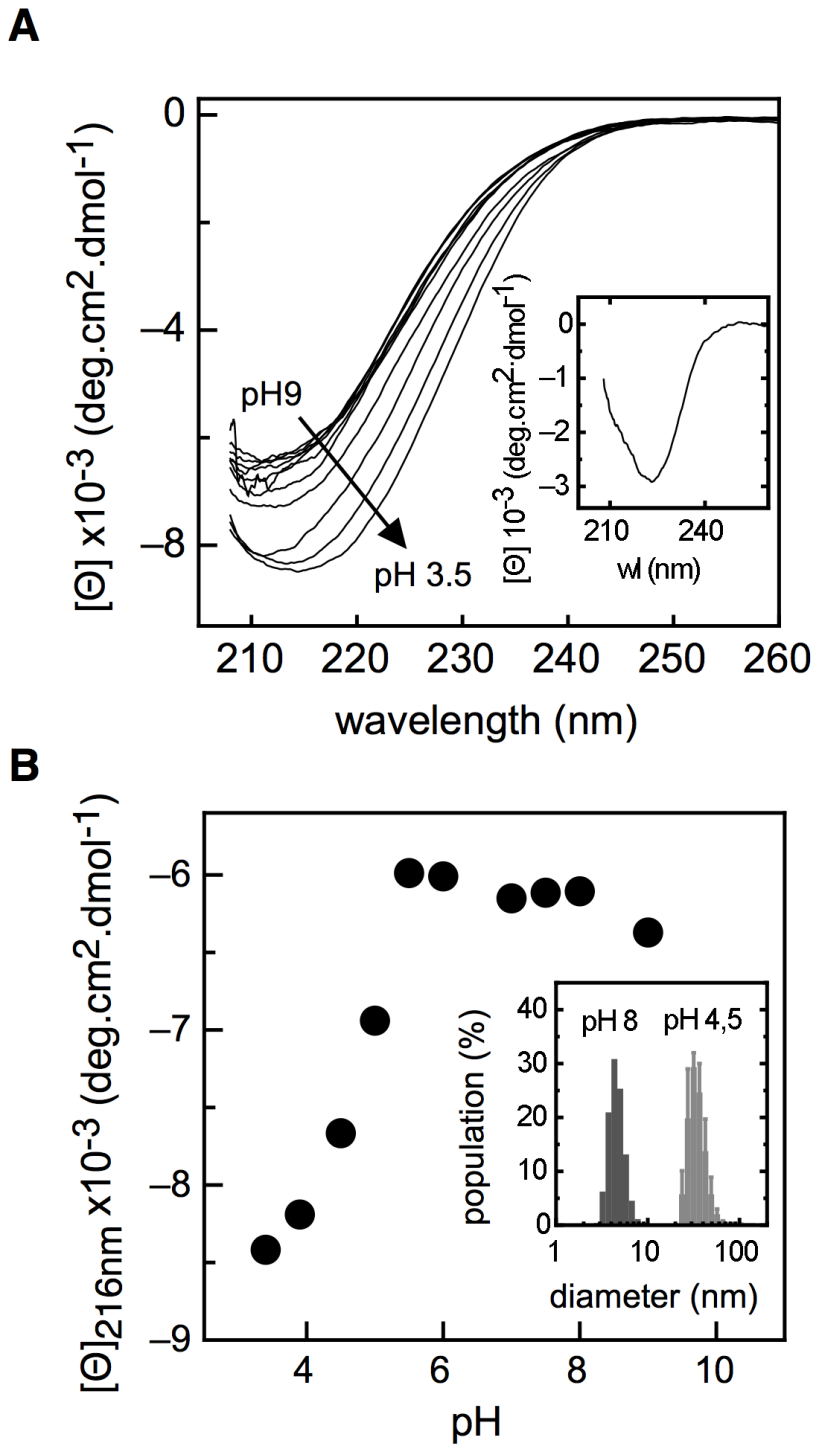
pH induced conformations. (A) Far-UV molar ellipticity measurements at different pHs. The inset shows the difference spectra between spectrum at pH 8 and pH 3,5. (B) Far-UV molar ellipticity at 216 nm between different pH values (•). The inset shows the particle size distribution of NS1 at pH 8 and pH 4.5. Experiments were carried out in a broad range buffer (20 mM Tris-HCl, 10 mM sodium acetate, 10 mM MES and 1 mM DTT) at 20°C.

Next, we evaluated the conformational transition of NS1 across a pH range from 3.5 to 9.0. For this, we first analysed the change in secondary structure by far-UV CD, shown in Figure 2A. As long as the pH decreases, the spectrum gradually changed its wavelength minima and increased the negative value for ellipticity, which appears to be, at least in part, to a minor increase in α-helical content but principally to a higher content in ß-sheet secondary structure, as strongly suggested by the difference spectrum (Fig. 2A, inset, [27]). Analysis of the transition by the molar ellipticity change at 216 nm showed a sharp drop in the ellipticity, coincident with its theoretical pI (5.7); however, no obvious insoluble aggregation was observed. The fluorescence intensity and CSM showed little or no changes across the pH range, which indicates a persistent tertiary structure and a still buried tryptophan (not shown). Both probes indicate that NS1 is not only very stable to pH denaturation, but also the DLS analysis showed that the species at low pH are soluble oligomers, based on the increase of hydrodynamic diameter from 4,2±1,2 nm to 32,7±3 nm (Figure 2B, inset). The process was not reversible.

### Soluble Spherical Oligomers Triggered by Temperature Increase Show Amyloid-like Properties

**Figure 3:**
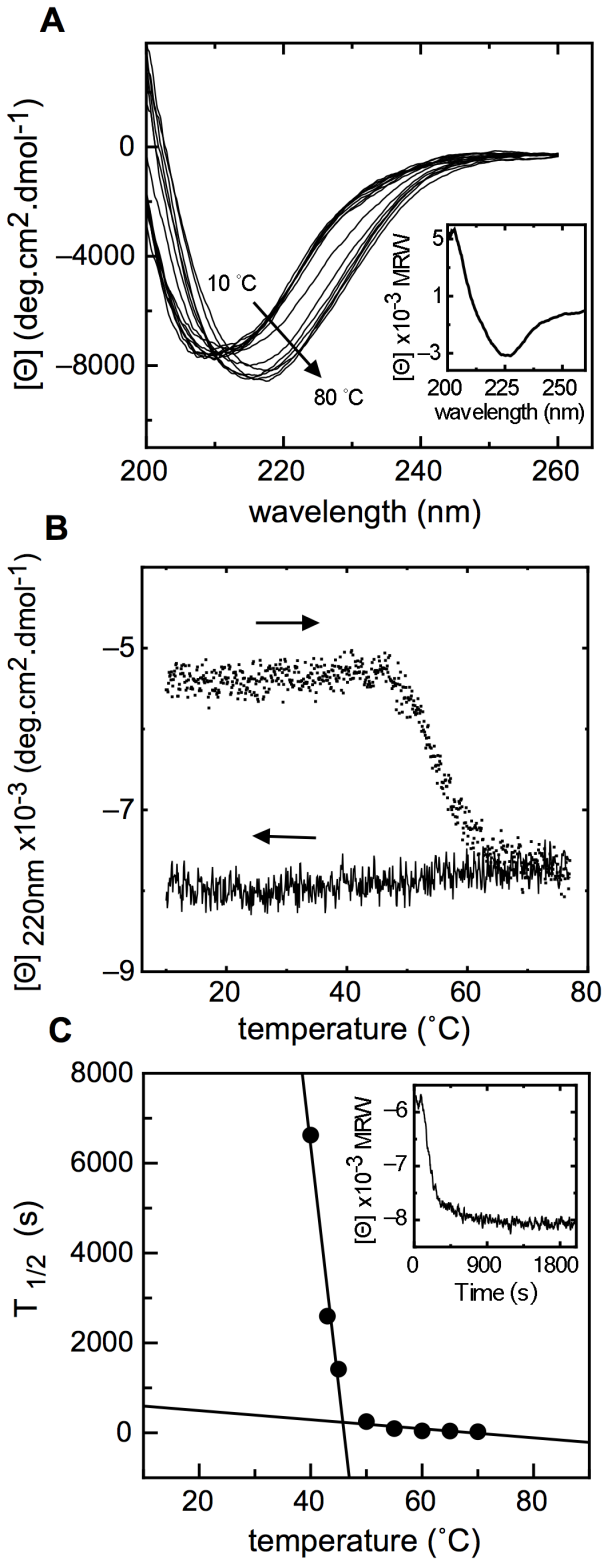
Thermal induced conformations. (A) Far-UV CD thermal denaturation of NS1 starting from 10°C to 80°C with a scan rate of 5°C/min. The inset shows the difference spectra between the heated and unheated species. (B) Thermal scans of heated and cooled sample followed at 220 nm of the far-UV CD spectra showing the irreversibility of the process. (C) Temperature-dependent kinetics: the half time of each main phase rate adjusted to a single exponential decay versus temperature are shown. The temperature-dependent kinetics performed at 55°C is represented in the inset.

We investigated the effect of temperature, as a mean to address the conformational stability of NS1, in particular at ranges compatible with biological activity. We analyzed changes in secondary structure by recording far-UV CD spectra at different increasing temperatures. There was an increase in negative ellipticity and a clear shift from ∼210 nm to ∼216 nm, suggesting an increase in the ß-sheet content, and resembling that caused by pH (Figure 3A and inset). An important conclusion is that the protein not only did not unfold at high temperatures but gained secondary structure content. The cooperative transition started at 45°C, showed an apparent T_m_ of 55°C, and was completed at 65°C, but the process was completely irreversible (Figure 3B). However, there was no visible precipitate and no decrease in protein concentration after centrifugation, indicating that the species formed are fully soluble and thermostable in solution. As an oligomer was also formed by lowering pH, we measured the size of the thermally “denatured” species that eluted at the exclusion volume of a size exclusion chromatography, indicative of its oligomeric nature (see below). The required temperature at the beginning of the transition was rather low and within the biological range, but the temperature scans, were carried out at relatively fast, nevertheless identical, rate intervals. Thus, we tackled the kinetic analysis of the temperature effect by carrying out temperature jumps monitored by ellipticity changes at 220 nm, in order to lower noise (Figure 3C, inset). The half-life for each jump trace was plotted against temperature. A very sharp brake was observed in the linear dependence, where the intercept of the two lines occurred at 45°C.

**Figure 4:**
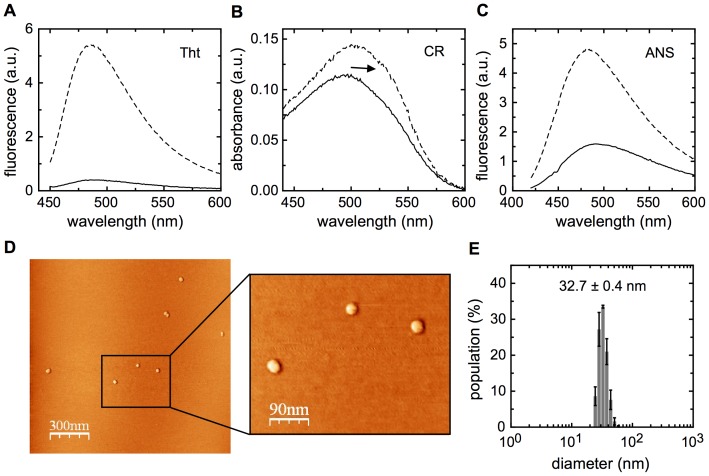
Structural and binding properties of NS1SOs. (A, B and C) Thioflavin T (Tht), Congo Red (CR) and 8-anilino-1-naphthalenesulfonic acid (ANS) binding of NS1SOs. Monomeric protein was measured before and after temperature treatment to form NS1SOs (full and dashed lines, respectively). The concentrations used were 20 µM for Tht, 100 µM and 5 µM for CR. (D) Atomic force microscopy: NS1SOs absorbed onto a freshly cleaved mica under 10 mM buffer Tris pH 8, and 4 mM MgCl2 (see methods). (E) Hydrodynamic diameter measured by DLS.

Further characterization of the temperature denatured oligomeric NS1, included the analysis of differential binding of thioflavin T (ThT) and Congo red (CR), typical of amyloid-like or repetitive ß-sheet properties. A large increase in ThT fluorescence and an increase and shift in the absorbance maximum for CR (Figure 4, top panel) are indicative of soluble amyloid-like or repetitive ß-sheet structures. A large increment of ANS fluorescence was also observed (Figure 4, C), which indicates a gain in solvent accessibility to hydrophobic structures in the oligomer.

Next, we sought to determine NS1 oligomer molecular size and shape. We saw that the oligomer corresponded to a monodisperse species with a diameter of 32.7±0.4 nm as judged by DLS (Figure 4, E). AFM showed the presence of regular spherical particles (Figure 4, D), and a statistical analysis of the particles yielded a diameter of 20.6±2.5 nm, in agreement with what was observed by DLS, considering different techniques. The discrepancy likely comes from the fact that DLS measurement is made in solution and calculates an average of all the particles present, while the AFM is not determined in solution and anomalous larger sizes are left out from the analysis. As a result, DLS yielded a larger value. The particles were indeed spherical and from now on we refer to them as NS1 spherical oligomers, NS1SOs, following nomenclature used in several other systems we previously characterized [28], [29], [30], [31].

### Comparative Conformational Stability and Unfolding of NS1 and NS1SOs

For the analysis of conformational stability, the NS1 monomer was chemically perturbed by unfolding with Gdm.Cl. Based on the spectral changes of folded and unfolded species (Figure 1A and 1B), the process was monitored by following changes in molar ellipticity and CSM.

**Figure 5:**
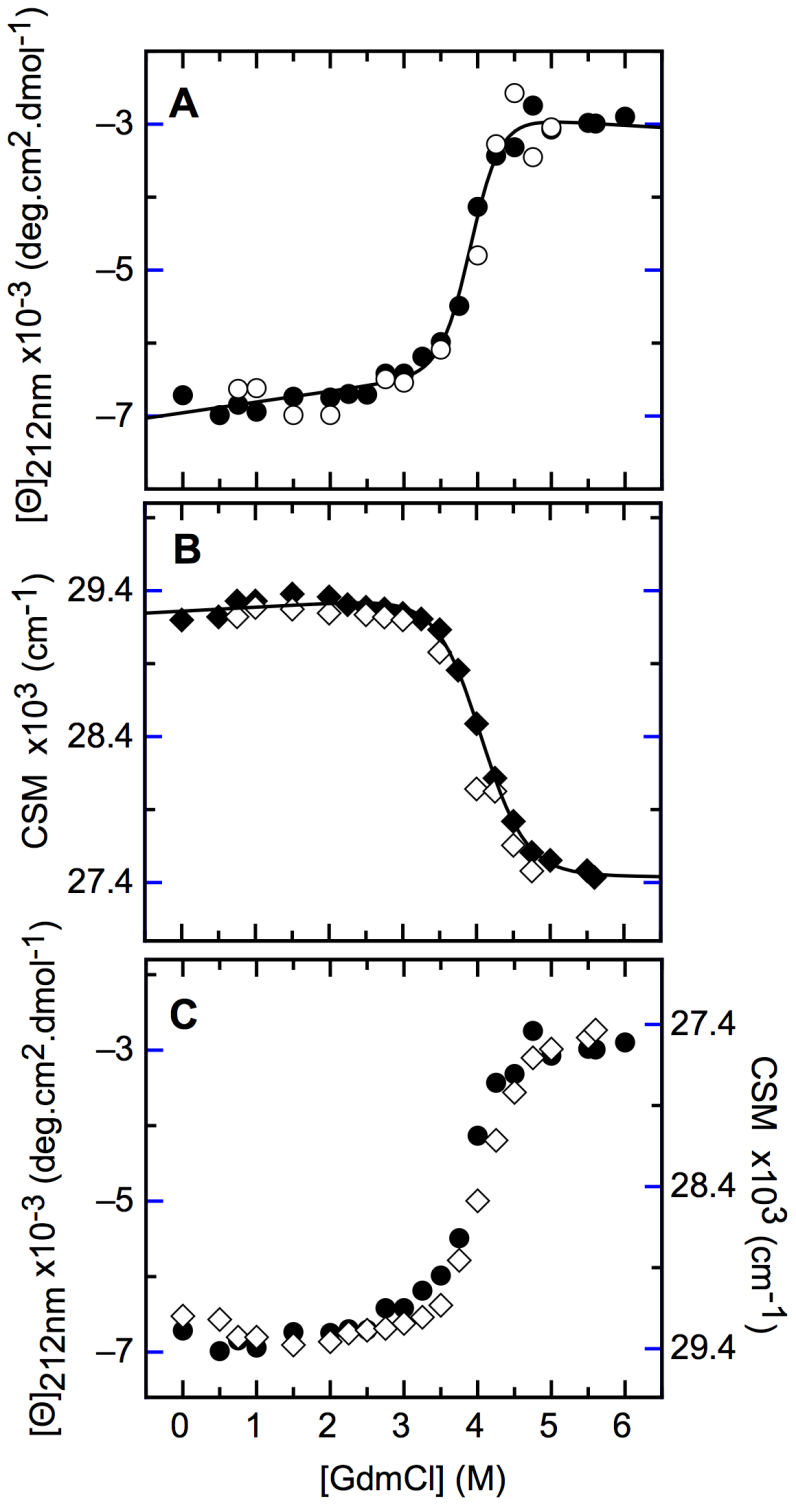
Gdm.Cl-induced denaturation performed at equilibrium. (A) Far UV CD molar ellipticity of unfolding (black circles) and refolding (white circles) experiments. (B) Fluorescence center of spectral mass (CSM) obtained from unfolding experiments (black diamonds) and refolding experiments (white diamonds). The continuous line in A and B was obtained after fitting data to a two-state denaturation model. (C) Far-UV CD signals superimposed to fluorescence signals (note that CSM scale is inverted). All measurements were carried out using a protein concentration of 10 µM.

NS1 unfolding was through a single highly cooperative transition (Figure 5A) and the ellipticity changes took place concomitantly with those of CSM, indicating that secondary and tertiary structure were highly coupled (Figure 5C). The gradual refolding was indicative of a fully reversible process (Figures 5A and 5B, white symbols). A global fitting of the data to a single monomeric transition (see methods) yielded a free energy change of 9.6±0.9 kcal·mol^−1^. The m value, the cooperativity indicator for the transition, was 2.5 kcal·mol^−1^·M^−1^. Overall these results confirm a two-state transition at the equilibrium.

**Figure 6:**
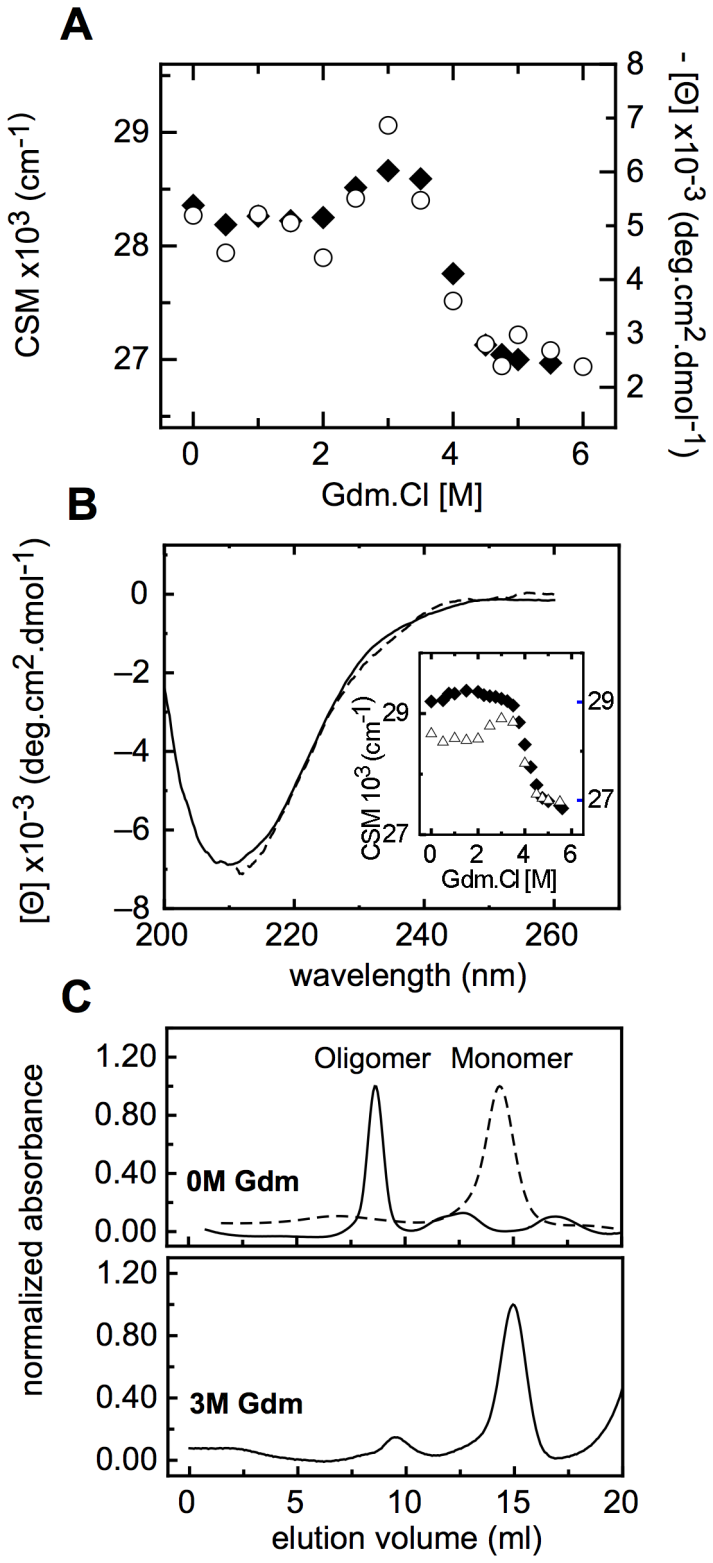
NS1SOs Gdm.Cl induced denaturation. (A) The left axis represents the fluorescence CSM (black diamonds) and the right axis the far-UV CD molar ellipticity at 215 nm (white circles), note that the scale of the latest is inverted. (B) Far-UV CD spectra of native NS1 (full line) and the dissociated NS1SOs intermediate in 3 M Gdm.Cl (dashed line). The inset shows the Gdm.Cl induced unfolding of monomeric NS1 (black diamonds) superimposed to the Gdm.Cl induced unfolding of NS1SOs (white triangles) (C) Size-exclusion chromatography of NS1SOs (full line) and NS1 monomer as a control (dashed line), see methods. Each conformer was incubated in 0 and 3 M of Gdm.Cl for 2 hs. The void volume of the column was around 8.8 ml and the expected elution volume for a 15.5 KDa protein according to a calibration with protein standards is around 14.5 ml. Note that monomeric NS1 in 3 M Gdm.Cl displays the same elution profile as native protein (not shown).

In order to evaluate the stability of NS1SOs, we carried out a similar Gdm.Cl denaturation experiment, starting from NS1SOs produced by heat treatment (Fig. 3A). In this case, two distinguishable transitions were observed, where ellipticity and fluorescence changes occurred concomitantly (Figure 6A). The first transition involved an increase in the CSM value, suggestive of an increased burial of the single tryptophan residue, and a small gain of negative ellipticity that, suggests a gain in secondary structure content. However, the change may well be qualitative rather than quantitative. The second transition showed changes that indicate full unfolding, i.e., exposure of the tryptophan to the solvent, and loss of secondary structure (Figure 6A). If we superimpose the CSM from the second unfolding transition of the NS1SOs to that of the NS1 monomer, the coincidence is remarkable, strongly suggesting that this transition corresponds to the unfolding of a dissociated native-like monomer (Figure 6B, inset). To corroborate this, we carried out a size exclusion chromatography experiment at 3.0 M Gdm.Cl (Figure 6C), where we observed that the species was largely monomeric, confirming the hypothesis of the dissociation of the NS1SOs to a native-like NS1 monomer, and its subsequent unfolding. Superposition of the Far-UV CD spectra of native and NS1SOs-dissociated monomer at 3.0 M Gdm.Cl, further supports the hypothesis that they are the same species (figure 6B).

### Different Solvents Converge in the Formation of ß-sheet Enriched Oligomers

**Figure 7:**
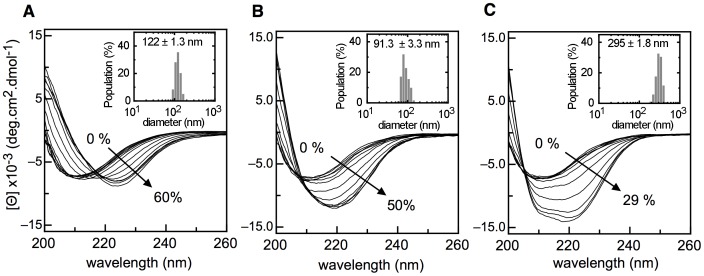
Cosolvents effects on NS1 conformation. (A, B, C) Effects of 2-propanol, ethanol and TFE titrations, respectively, are shown. The percentage of initial and final solvent concentration is indicated. The inset for A, B and C panels show the DLS measurements after incubating NS1 for 1 hour with 30% 2-propanol, 50% ethanol and 15% TFE, respectively.

Given that temperature and pH shift the equilibrium towards stable, soluble oligomers, we wanted to investigate the effect of solvent perturbations. We chose solvents of different ranges of dielectric constant, known to stabilize pre-existing structures [32]. Titration with increasing amounts of ethanol and isopropanol, led to a shifted ß-sheet enriched structure (Figure 7A and 7B). Trifluoroethanol also underwent a similar shift to some degree, but it was subsequently driven to the formation of α-helix (Figure 7C). Eventually, the three solvents stabilized α-helix secondary structures to some extent (not shown), but TFE is well known to be one with the strongest effect. In agreement with this, high percentage (80%) of methanol was required to cause the same effect (not shown).

**Figure 8:**
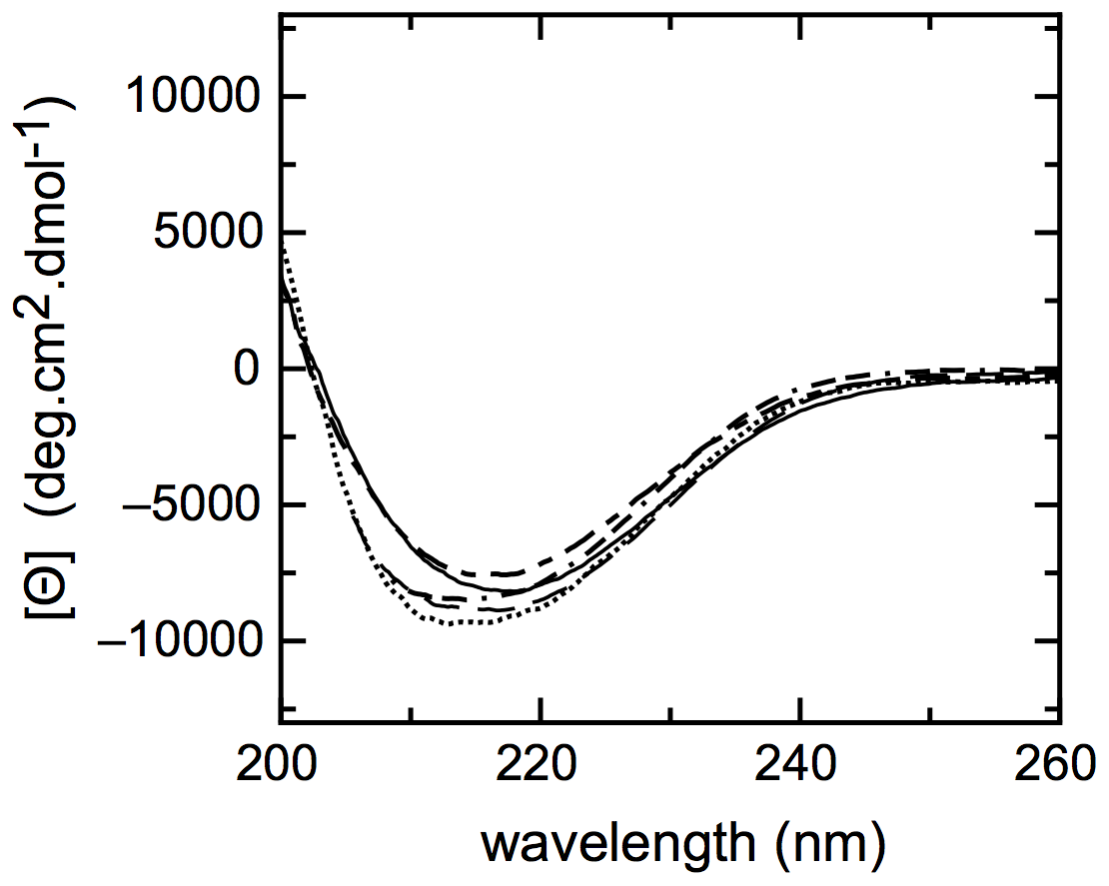
Conformational changes triggered by the different conditions tested. Far-UV CD spectra of temperature induced NS1SOs (−) and NS1 incubated at pH 3.5 (−− ⋅ −−). The spectrum of NS1 with 33% ethanol (− − − −), 28% 2-propanol (………) and 15% TFE (− − −) are shown.

Finally, we determined the molecularity and size of the species at solvent concentrations in which they form the ß-sheet increased species, by DLS. Monodisperse species of 122±1.3, 91.3±3.3, and 295±1.8 nm diameter for ethanol, isopropanol, and TFE, respectively, were observed. We can conclude that in the three solvents tested, stable and soluble oligomers were formed, but those in TFE corresponded to a larger size. We compared the far-UV CD spectra of the species under different conditions and they were highly superimposable (Figure 8).

## Discussion

Immune evasion of hRSV largely relies on the action of its non-structural proteins. Among several features that make NS1 from hRSV an intriguing protein, we should stress the fact that: i) NS1 is present in RSV and not in all other members of the paramyxovirus family [1], ii) NS1 interacts with a large list of host proteins [24], [25], iii) there is an absence of any tract of sequence homology in genome databases. In order to dissect the molecular mechanism behind NS1 possible function or effects in infected cells, we should start investigating its biochemical behaviour. The information in this respect is scarce, and there is no structure to guide the analysis of the conformational equilibria and interactions with host proteins. Therefore, in this study we investigated the conformational properties and thermodynamic stability of NS1 making use of rational chemical and physical perturbation.

The first indication of high stability comes from changes induced by pH. Lowering pH up to 3.0 not only does not unfold the protein, but also increases its secondary structure content in the form of an irreversible and soluble oligomer (Figure 2). The transition midpoint around pH 5.0 suggests that a monomeric form may not be the only conformation present within cells.

Temperature increase also leads to the irreversible formation of soluble oligomers with increased ß-sheet content. These oligomers are similar in diameter (32.7±0.4 nm) to those induced by pH (32.7±3 nm), and further characterization by AFM shows a monodisperse spherical shape. In addition, NS1SOs bind to dyes characteristic of amyloid like repetitive ß-sheet structures. Interestingly, despite its stability and based on irreversibility and increased secondary structure, they bind ANS, which is indicative of solvent accessible hydrophobic cavities, therefore suggesting a less packed tertiary structure. Based on NS1SOs globular nature we can estimate, from a linear calibration curve [33], that their diameter corresponds to a 2.3 MDa species, which represents 149±4 NS1 monomers per molecule of oligomer.

An onset of ∼45°C for the transition to NS1SOs is compatible with physiological values. We found that a kinetic analysis is more appropriate and accurate for quantitative assessment. Remarkably, the half-lives increase by 25-fold in going from 40 to 46°C, corresponding to the apparent Tm obtained from the kinetic analysis. Further, the irreversible nature of this process indicates that even slow changes inevitably lead to the gradual accumulation of oligomeric species at temperature conditions compatible with those within cells. It should be taken into consideration that an exact value can never be obtained for a process in vivo, this will vary according to numerous factors such as crowding, interaction with other proteins and so forth. The relevant fact is that the temperature conditions are not harsh and lie within the physiological range.

Fully reversible chemical denaturation by Gdm.Cl allows for the characterization of a clear-cut two-state transition to the unfolded state, and the stability of the NS1 monomer. The process is highly cooperative and completely reversible. For a set of model proteins, the average free energy of unfolding is Δ*G^0^/N*, is 0.075±0.037 kcal·mol^−1^.res^−1^, where N corresponds to the protein number of residues and no detectable correlation is observed between size and normalized stability [34]. The value for NS1, 0.069 kcal·mol^−1^ is within this range.

The analysis of the unfolding of the NS1SOs involves a first transition to the monomer (2.0 to 3.0 M Gdm.Cl), suggesting, a dissociation to a species that not only bears secondary structure and hydrodynamic properties identical to the native monomer, but also presents a superimposable unfolding transition to the final unfolding state (Fig. 6). These results indicate that an energy barrier must be overcome for the irreversible formation of NS1SOs; this corresponds to a conformational change leading to the monomer that is the subunit of the oligomers. Nevertheless, this process can be reversed by Gdm.Cl, where the “unfolding” energy barrier implies dissociation and recovery of the native monomer conformation prior to unfolding. Further analysis of kinetic mechanisms will be required to understand the overall process and dissect the order of the events.

Decreasing the polarity of the milieu also shifts the equilibrium to oligomers with CD spectra resembling that of NS1SOs induced by temperature, suggesting a common route and endpoint (Figure S1). In the case of isopropanol and ethanol, these solvents are usually more likely to stabilize α-helix, which means that the formation of oligomers through the increase of ß-sheet content is a strong tendency of the system itself.

Many proteins may form amyloid-like structures given the appropriate conditions, but some others show a stronger tendency based on their amino acid composition [35], [36]. Some of these proteins are well known to form amyloids and to be related to human disease such as the amyloid Aβ peptide, β2-microglobulin, transthyretin and lysozyme proteins, and others [37], [38], [39], [40], [41]. However this structure is not only related to misfolding diseases and could be formed in functional procceses such as the case of native Pmel17 amyloid in mammals [42]. In addition, we have previously reported viral proteins that form amyloid structures as the E7 oncoprotein, the DNA binding C-terminal domain E2C both from Human Papillomavirus and the DNA binding domain of the nuclear antigen 1 (EBNA1) from Epstein-Barr Virus [28], [29], [30]. In the case of E7, we showed that E7SOs localized to the cytosol in different eukaryotic cell lines and in cells from tissue biopsies, while E7 in the dimeric and monomeric forms are localized in the nucleus and are replenished dynamically from the cytosolic pool [43]. Moreover, the negative sense RNA virus, Influenza A, encondes the PBI-F2, which has been shown to form amyloids under hydrophobic conditions and to form different types of oligomers and fibers in infected cells in the vicinity of cellular membranes [44]. As NS1 also forms oligomeric structures under similar conditions and as it was reported to be associated to the mitochondria in infected cells, the formation of these amyloid structures could be related to NS1 function in this subcellular localization as in PBI-F2 protein.

A comparison of NS1 primary sequence with its bovine and murine homologs revealed a 69% and 16% of amino acid sequence identity between hRSVA and Bovine RSV and Pneumonia Virus of Mice (PVM) [1]. Recently, Elliott et al., [45], showed that NS1 contains elongin C and cullin 2 binding consensus sequences and is able to interact with elongin C and Cul members to form an active E3 ligase complex to downregulate STAT2 activity, a similar mechanism reported for other viral proteins. However, this consensus sequence is not present in PVM and not other sequence homologies have been found that could explain NS protein functions. These observations may suggest that although human and bovine counterparts share high sequence homology, there is a low sequence homology with the murine virus and this could reflect that they may share structural motifs rather than sequence motifs that could be similar to other paramyxovirus IFN antagonists.

The fact that NS1 is a small protein with multiple interacting partners described must necessarily be related to conformational diversity. In this work we show that NS1 conformation is highly dependant on environment conditions such as pH, hydrophobicity and temperature that are within physiological ranges. Structural heterogeneity is compatible with a complex cellular environment affected or modulated by crowding, specific protein-protein interactions, and subcellular localization [46], [47].

As we mentioned, NS1 co-localizes with the mitochondria associated protein MAVS. It was recently reported that MAVS forms large aggregates after viral infection and these species form fiber polymers with amyloid properties, which act as functional aggregates as they are highly potent in activating IRF3 [48]. NS1SOs may interact with MAVS oligomers, preventing the formation of these fibrilar functional aggregates to block downstream signaling. Identification of NS1SOs in infected cells and its relation to MAVS will be important to verify this hypothesis. In addition, NS2 was shown to interact with NS1, but it could not be determined whether this is a direct or an indirect interaction. It is likely that they form part of multisubunit complexes both in the IFN induction pathway, interacting with MAVS and RIG-I, and in the response pathway, forming part of an E3 ubiquitin ligase complex.

Further analysis will be required in order to dissect the folding mechanism and to define the role of the NS1 monomer and NS1SOs in infected cells. The development of specific antibodies will be a powerful tool to discriminate between each conformer and to study the specific localization within cells. Ultimately, this will lead to underlying molecular mechanisms related to hRSV evasion of interferon-mediated innate immunity.

## Materials and Methods

### Protein Expression and Purification

The human RSV strain A NS1 sequence was cloned into the BamHI and HindIII sites of the pMal vector, which expresses NS1 fused to the maltose binding protein (MBP) with a thrombin cleavage sequence linker. The construct was transformed into C41 cells and expression was induced by the addition of 0.3 mM IPTG at 20°C at ≈ 0.6 of OD. Cells were harvested by centrifugation 18 h after induction and stored at −70°C. Cell pellet was resuspended in 50 mL of buffer containing 20 mM Tris-HCl pH 8.0, 0.2 M NaCl, 5 mM 2-mercaptoethanol, and 1 mM EDTA, lysed by sonication, and centrifuged for 30 min at 15000 *g* at 4°C. The resulting supernatant was precipitated via addition of solid ammonium sulphate to 50% saturation. The precipitated protein was collected by centrifugation, resuspended, and dialyzed against 10 mM Tris-HCl pH 8, 0.2 M NaCl, and 2 mM 2-mercaptoethanol buffer. MBP–NS1 fusion protein was enriched from the soluble bacterial fraction using an amylose resin (New England Biolabs, Hitchin, UK) and MBP was cleaved by treatment with thrombin (Sigma-Aldrich, St Louis, MO, USA) using 0.25 NIH standard units per 3 mg of protein. NS1 was separated from MBP by a Superdex-75 gel filtration column. Then, the NS1 fraction was dialyzed against 10 mM Tris pH 8.0, 0.2 M NaCl, and 1 mM DTT buffer. This procedure yielded ≈ 10 mg of >95% pure NS1 per liter. Protein purity was judged by SDS-PAGE and protein identity confirmed by western blots and MALDI-TOF MS (Bruker, Daltonics, Billerica, MA, USA).

NS1SOs were generated by incubating for 10 minutes at 70°C in buffer Tris 10 mM pH 8 and DTT 1 mM.

### Size Exclusion Chromatography

Size exclusion chromatography experiments were carried out on a Superdex 75 HR 10/30 (24 mL). The column was calibrated with ovalbumin (44,2 KDa), chymotrypsinogen A (25 KDa), and ribonuclease A (13.7 kDa). The void volume (V0) and total volume (Vt) were determined by loading Blue Dextran and acetone, respectively. The buffer used was Tris 10 mM pH 8 and DTT 1 mM.

### Static Light Scattering

The average molecular weight of NS1, was determined by static light scattering (SLS) using a Precision detector PD 2010 light scattering instrument connected in tandem to a high-performance liquid chromatography system and a LKB 2142 differential refractometer. The 90° light scattering and refractive index signals of eluted protein were recorded on a personal computer and analyzed with the Discovery32 software supplied by Precision Detectors. The protein concentration used was 190 µM and 200 µl was used for each run. The 90° light scattering detector was calibrated using bovine serum albumin (Mw: 66.5 KDa) as a standard.

### Dynamic Light Scattering

DLS measurements were carried out on Zetasizer Nano S DLS device from Malvern Instruments (Malvern). NS1 was filtrated with Ultrafree-MC 0.22 mm microcentrifuge filters (Millipore) before measurements were done. NS1 protein concentration was kept between 20 and 40 uM, and temperature was maintained at 20’C by Peltier control system.

### Spectroscopy

*Circular Dicroism*. Far-UV CD measurements were carried out on a Jasco J-810 spectropolarimeter using a Peltier temperature-controlled sample holder at 20°C in a 0.1 cm-path-length cell. Spectra between 190 and 260 nm were recorded at a rate of 100 nm/min, a response of 2 s and a bandwidth of 2 nm. All spectra were an average of at least four to six scans. The results were expressed as degrees per square centimetre per dmol.

*Fluorescence*. Experiments were performed in a Jasco FP-6500 spectrofluorometer. For equilibrium measurements Trp fluorescence emission spectra were recorded between 310 and 450 nm with excitation wavelength of 295 nm. Tht and ANS fluorescence were performed with an emission spectra wavelength between 420 and 600 nm with an excitation wavelength of 442 nm for Tht and 370 nm for ANS, respectively. Fluorescence data were analyzed after subtracting the buffer background composition.

For monitoring transitions, we used the Center of Spectral Mass (CSM) that is calculated by the following equation:
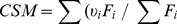
(1)where F_i_ is the fluorescence emitted at wavenumber υ_i._

The data from Gdm.Cl unfolding and refolding experiments were fitted to a two-state denaturation model, and parameters were determined by a non-linear curve fitting to the following equation:
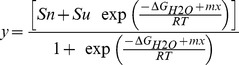
(2)where y is the observed spectroscopic signal; S_n_ and S_u_ represent the spectroscopic signals of folded and unfolded proteins, respectively; ΔG and m represent the free-energy change and slope of the transition, respectively; x is the denaturant concentration; T is the temperature (in K), and R is the universal gas constant (1.987 kcal/mol).

### Atomic Force Microscopy

Experiments were performed in a Nanoscope III Multimode-AFM (digital Instruments, Veeco Metrology, Santa Barbara,CA) using a Tapping mode. NS1 SOs were diluted to 5 ng/µL in Tris 10 mM pH 8 and 4 mM MgCl_2_. Five µl of sample were deposited onto freshly cleaved muscovite mica. After 5 min, the sample was gently washed with 1 ml milli-Q water to remove molecules that were not firmly attached to the mica and blown dried with nitrogen. Images were processed by flattering using Nanoscope software (Digital Instruments) to remove background noise. Images were analyzed with a WsxM 4.0 beta 5.3 software.

## Supporting Information

Figure S1 Cosolvent titrations. Far-UV CD molar ellipticity at 220 nm for different alcohol titrations: ethanol (black circles), 2-propanol (white diamonds) and TFE (white circles).
(TIF)
