# Supplementary figures and images for: The Non-Structural NS1 Protein Unique to Respiratory Syncytial Virus: A Two-State Folding Monomer in Quasi-Equilibrium with a Stable Spherical Oligomer
Source: PLoS One. 2013 Sep 10;8(9):e74338. doi: 10.1371/journal.pone.0074338 (PMC3769240; doi:10.1371/journal.pone.0074338)

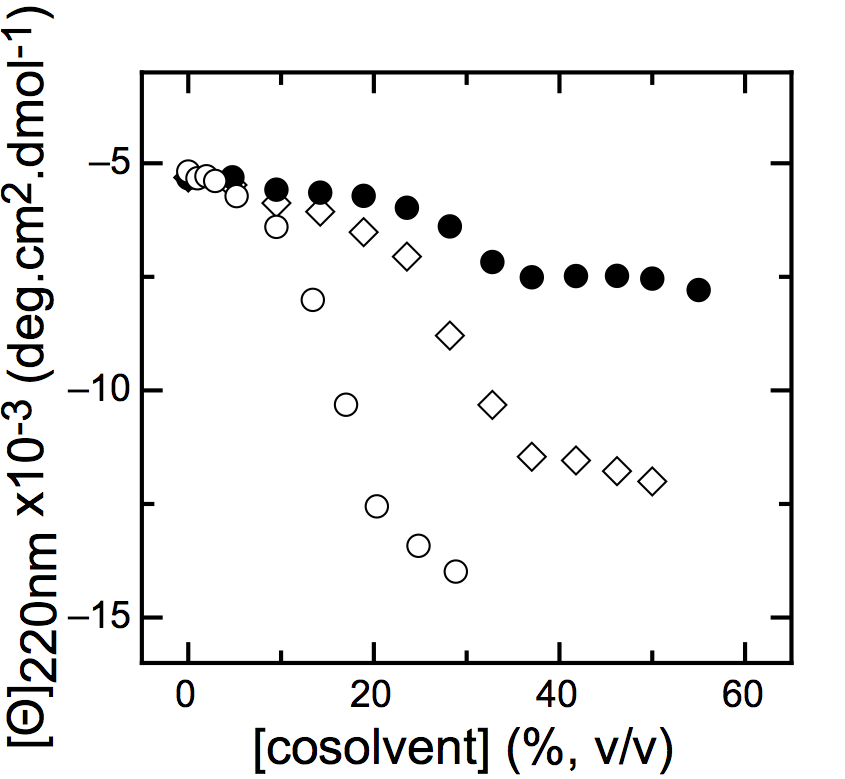

Supplement: Figure S1 — Cosolvent titrations. Far-UV CD molar ellipticity at 220 nm for different alcohol titrations: ethanol (black circles), 2-propanol (white diamonds) and TFE (white circles). (TIF) [file pone.0074338.s001.tif]
